# Mycosis Fungoides: Analysis of Ophthalmologic Findings in a Series of Cases

**DOI:** 10.1155/2019/2380598

**Published:** 2019-01-20

**Authors:** Carina G. Colossi, Juliano Mondadori, Pedro K. M. Barreto, Felipe M. Valença, Rodrigo Duquia, Manuel A. P. Vilela

**Affiliations:** ^1^Institute of Cardiology/University Cardiology Foundation and Post-Graduation Program, Ivo Corrêa-Meyer Institute of Ophthalmology, Porto Alegre-RS, Brazil; ^2^Federal University of Health Sciences of Porto Alegre, Brazil

## Abstract

**Background:**

Ophthalmic findings in mycosis fungoides (MF) can be highly variable. It seems that the prevalence of ophthalmic findings could be much more common than previously assumed.

**Objective:**

To present case series examined in the last 12 months, together with a literature review.

**Methods:**

Symptomatic patients with biopsy-proven mycosis fungoides were examined ophthalmologically in a 12-month period. The medical records of affected patients were reviewed.

**Results:**

Eight patients were examined. Of these, 75% were male, all were Caucasian, and average age was 58.2 years. Blepharitis (50.0%), thickened eyelids (37.5%), and flaking (25.0%) were the most prevalent findings.

**Conclusion:**

Incidence of MF affecting the eyes and surrounding structures may be greater than estimated. Early case management offers means to reduce difficulties experienced with later diagnosis. Regular monitoring by an ophthalmologist is justified, including that of asymptomatic cases.

## 1. Introduction

Mycosis fungoides (MF) is a rare primary T-cell cutaneous lymphoma. Its annual estimated incidence is 6.4-9.6 cases per one million inhabitants in the USA [1–3]. Clinical diagnosis is usually difficult, especially at early stages. Diagnosis often requires multiple biopsies and is commonly performed quite late, given its similarities with other conditions and the scarcity of typical clinical or histopathologic signs [2–5]. Blotches or flaking erythematous plaques are usually the initial manifestation, making the condition similar to other skin disorders such as eczemas, atopic dermatitis, psoriasis, and pityriasis lichenoides. In the next stage infiltrative lesions appear as the disease progresses. Following this, tumorous lesions appear and lymph nodes and other organs are affected. It is a disease mostly affecting White adults. As a rule the disease is indolent and controllable. Available treatment is for the most part palliative, although it is free from risks and serious side effects and improves long-term control and survival [1–5]. Bone marrow transplant is the only treatment that cures the disease but is rarely adopted [6, 7].

MF involving the eyes and their surrounding structures is considered to be rare (2% of affected cases) and is probably underestimated owing to difficulties in diagnosing it in the early stages [1]. Only three sizeable series can be found in the accounts available in the literature. Among the 30 patients described by Stenson & Ramsay [8], 36.7% had ophthalmological changes and eyelid tumours were the most common finding (26.7%). Leib et al. [9] described 17 cases and eyelids were the most frequently affected region by blepharitis or ectropion. Cook et al. [10] assessed 210 cases, whereby the most prevalent condition was ectropion (40.4%).

Our aim is to present case series examined over the last 12 months, to review the literature, updating manifestation in the eyes and their surrounding structures, as well as possibilities for case management.

## 2. Case Report

In the period comprising October 2016 to October 2017, eight cases with clinical and histopathologic diagnosis of MF were referred by the Dermatology Service of a University in Southern Brazil. These were only visually symptomatic patients and did not account for all patients examined in that period (total number of cases with MF: 40). The ophthalmological examination consisted of corrected Snellen visual acuity, extrinsic motricity, pupil reflexes, applanation tonometry (Perkins), anterior and posterior biomicroscopy (Topcon SL 1E, Tokyo, Japan and 90-D Volk lens, USA), and indirect binocular ophthalmoscopy with mydriasis (Eyetec model ODS 6.0, São Paulo, Brazil). All eight cases were examined. Average age was 58.2 years (29–77), the majority (75.0%) were male and 100% were Caucasian. All patients examined signed a free and informed consent form and this study was approved by the institution's Research Ethics Committee. Burning eyes (50%), progressive impaired vision (37.5%), and redness (37.5%) were the most common symptoms, followed by itchy, watery, and crusts on eyelashes and eyebrows (25%). Average corrected visual acuity was 0.90 in both eyes. Findings directly linked to MF were blepharitis (50.0%), eyelid thickening (37.5%), flaking skin (25.0%), and filamentary keratitis (12.5%). Age-associated findings were cataract (37.5%), pingueculae (37.5%), and macular RPE changes (12.5%). 62.5% were undergoing phototherapy (PUVB) whilst the remainder were only receiving symptomatic maintenance therapy. Demographic characteristics and ophthalmic findings are shown in Table 1.

## 3. Discussion

MF can be the result of chronic antigenic stimulation, possibly owing to occupational or infectious factors or genetic mutations, triggering uncontrolled clonal growth and accumulation of T helper lymphocytes on the skin. It usually progresses slowly and 70% of cases are diagnosed at this stage, having erythematous plaques distributed in regions of the body usually protected from the sun (buttocks, breasts, and lower torso). In 30% of cases the disease is more aggressive and forms infiltrative or tumorous lesions and in some cases tends to develop with erythroderma [2–5, 11].

In terms of histopathology, MF consists of multiplication of CD4^+^CD45RO T lymphocytes, with some cases of CD8^+^ expression (20%). Neoplastic cells are commonly atypical small or intermediate lymphocytes with hyperchromatic nuclei and having a cerebrum-like aspect surrounded by clear cytoplasm (10%). Infiltrate occurs along the dermoepidermal junction, with lymphocytic epidermotropism present in 96% of cases in the form of clusters or isolated cellularity, with no spongiosis in 25% of cases (Pautrier's microabscess). Specific cytopathic modifications, keratinocyte apoptosis, Civatte bodies, and colloid bodies also often occur. Molecular markers generally do not show positive cellularity enabling disease definition, especially in its initial stages. Presence of the TOX gene in the cells may assist confirmation of diagnosis. These difficulties have led to the creation of a diagnostic algorithm that integrates clinical, histologic, immunophenotypic, and molecular criteria by the International Society for Cutaneous Lymphomas [3–5, 12].

Estimated MF prevalence in the eyes and surrounding structures is low (2%) and among those having visual symptoms it is 26.7%–40.4% [7–11, 13, 14]. Incidence is possibly underestimated given the difficulty in diagnosing it and late diagnosis, in addition to clinical variants. Primary eye conditions, prior to skin repercussions, are extremely rare. The eyelids and eye surface are usually the most affected areas, particularly in more advanced stages. Ectropion is the most common manifestation on the eyelids, caused by tumours or cutaneous infiltration. Case management is complicated regardless of the mechanism and may compromise eye surface integrity [7–11, 13, 14].

In our series flaking blepharitis was the most common finding, followed by eyelid thickening and eyelash and eyebrow flaking. The most prevalent complaints were burning, impaired vision, redness, itching, and crust on eyelashes and eyebrows. The signs and symptoms found by Leib et al. [9] in their series were similar. Other regions directly affected by the process are the conjunctiva, sclera (episcleral infiltrate), corneas (punctuate keratitis, stromal opacification, melts, and subepithelial infiltrates), retina, choroid and vitreous body (infiltrates, uveitis, and vascular occlusions), and the optic nerve (infiltration and neuropathy) [8, 14–22].

Nonspecific manifestations include dry eyes, cataracts, corneal ulcers, glaucoma, chorioretinal scars, peripheral nerve palsy, and campimetric defects, among others. In our series, cataracts, exuberant pingueculae, and pigment change in the macular region were detected and associated with age. The most prevalent dermatological complaints were pruritus, erythematous-scaling plaques or rash on nonexposed areas, and infiltrative plaques.

Systemic case management is palliative. Evidence collected through systematic review concluded that monotherapy and different combinations showed similar results [23]. PUVA, in isolation or combined with interferon-alpha or retinoids, has been found to be beneficial in many situations [24]. PUVA may be a confounder, but all cases reported here used towels and goggles to protect the eye region. Systemic methotrexate or methotrexate associated with interferon-alpha or bexarotene in more advanced stages has produced favourable evidence. At local level, care of chronic eyelid edge infections, corneal exposure, and surface lubrication tend to be the most adopted measures. Eyelid thickening can be alleviated by using PUVA, but severe ectropion and tumours require surgical repair. Infiltrative conjunctival envelopment can be managed with radiotherapy and topical corticosteroids [16]. Choroid, retina, vitreous or optic disc infiltrations are associated guarded prognoses and may be treated with intravitreal methotrexate or other systemic chemotherapy drugs (5-fluorouracil, leucovorin) and/or localized radiation [25].

Our study is cross-sectional, small, and not representative of all MF cases we cared for during the period. Its strength lies in the description of ocular manifestations in cases that have not yet developed into visceral disease. Functional morbidity of the eyes at this stage is relevant and as such paying careful attention to patient complaints and early case management can reduce the severity of eye impairment.

In conclusion, the incidence of MF affecting the eyes and their surrounding structures may be greater than estimated. Early case management offers means to reduce difficulties experienced with later diagnosis. Regular monitoring by an ophthalmologist is justified and may contribute to reducing sequelae, including regular monitoring of asymptomatic cases when the likelihood of conversion to eye symptoms over time is still unknown.

## Figures and Tables

**Table 1:** MF case demographic, clinical, and examination characteristics.

Case	Age (years)	Sex	Clinical signs	Complaints	VA	Examination	General management
1	67	M	infiltrative plaques, scaly lesions	IMPAIRED VISION ITCHING, EYELASH CRUSTS, WATERY EYES	OD 0.7 OE 0.6	Eyelid thickening, blepharitis/conjunctivitis, eyelash and eyebrow flaking, filamentary keratitis, nuclear 2+ cataract	

2	77	M	erythema patches, pruritus	IMPAIRED VISION	OD 0.6 OE 0.6	Eyelid thickening, PSC cataract	

3	56	M	maculopapular rash and itchy lesions	BURNING	OD 1.0 OE 1.0	Exuberant pingueculae	PH

4	72	F	erythema patches, pruritus	BURNING IMPAIRED VISION	OD 0.6 OE 0.6	Exuberant pingueculae Mild axial cataract	PH

5	62	F	erythema patches, scale, pruritus	REDNESS; BURNING	OD 0.9 OE 0.9	Exuberant pingueculae, Macular RPE change	PH

6	29	M	Fine scale, itchy macules	REDNESS WATERY EYES	OD 1.0 OE 0.9	Blepharitis, eyelash flaking	

7	51	M	erythema patches, pruritus	ITCHING	OD 1.0 OE 1.0	Blepharitis	PH

8	52	M	maculo-papulomatous and itchy lesions	BURNING; CRUSTS, REDNESS	OD 1.0 OE 1.0	Blepharitis, eyelid thickening	PH

Average	58.2	M (75%)	Erythematous-scaling plaques or rash 87.5% Pruritus 62.5% Infiltrative plaques: 12%	BURNING 50% IMPAIRED VISION 37.5% REDNESS 37.5% ITCHING 25% CRUSTS 25% WATERY EYES 25%	OD 0.9 OD 0.9	Blepharitis 50% Cataract 37.5% Eyelid thickening 37.5% Pingueculae 37.5% Eyelash flaking 25.0% Filamentary keratitis 12.5% Macular RPE change 12.5%	PH 62.5%

M = male, F = female, VA = corrected visual acuity, PH = phototherapy, PSC = posterior subcapsular cataract, RPE = retinal pigment epithelium.
